# Assessing in house comprehensive genomic profiling by liquid biopsy for NSCLC patients

**DOI:** 10.1177/03008916261438383

**Published:** 2026-06-24

**Authors:** Andrea Vingiani, Alberta Piccolo, Adele Busico, Iolanda Capone, Elena Tamborini, Federica Perrone, Cinzia De Marco, Paolo Verderio, Chiara M. Ciniselli, Claudia Proto, Marta Brambilla, Giuseppe Lo Russo, Elena Conca, Andrea Devecchi, Daniele Lorenzini, Luca Agnelli, Giancarlo Pruneri

**Affiliations:** 1 Department of Diagnostic Innovation, Fondazione IRCCS Istituto Nazionale dei Tumori, Milan, Italy; 2 Department of Oncology and Hemato-Oncology, School of Medicine, University of Milan, Italy; 3 Bioinformatics and Biostatistics Unit, Fondazione IRCCS Istituto Nazionale dei Tumori, Milan, Italy; 4 Department of Medical Oncology, Fondazione IRCCS Istituto Nazionale dei Tumori, Milan, Italy

**Keywords:** biomarkers, comprehensive genomic profiling, liquid biopsy, ctDNA, non-small cell lung cancer, targeted therapy

## Abstract

**Purpose:**

Liquid biopsy has emerged as a valuable tool for detecting therapeutic targets and resistance mechanisms. This study evaluated the analytical performance and clinical utility of TruSight Oncology 500 ctDNA (TSO500) compared to the FDA-approved Guardant360 CDx (G360) in detecting actionable alterations in non-small cell lung cancer (NSCLC) patients progressing on targeted therapies.

**Patients and methods:**

We analyzed 44 plasma samples from 36 consecutive metastatic NSCLC patients with known molecular drivers (*EGFR* and *BRAF* mutations, *ALK* and *RET* fusions) progressing on targeted therapy. The comparative analysis included 31 paired samples from 27 patients. Valid results were obtained from G360 in 42/44 cases (95.45%) and TSO500 in 32/44 samples (72.8%).

**Results:**

TSO500 demonstrated high sensitivity for G360-identified variants (81.02% overall; 85.26% for pathogenic/likely pathogenic variants). Concordance was particularly high for ESCAT I alterations (95.2% sensitivity), including 100% sensitivity for gene fusions (five detected fusions). TSO500 identified 102 additional variants not detected by G360. Among nine patients with concurrent tissue biopsy, TSO500 detected 16 potentially actionable mutations absent in tissue samples. Both assays identified resistance mutations in 12/26 patients (46.2%), with TSO500 detecting all but three G360-identified alterations. High correlation was observed between variant allele frequencies (Pearson's r = 0.89).

**Conclusions:**

TSO500 demonstrates high concordance with G360 for detecting actionable alterations and robust fusion identification. Its ability to detect additional variants and resistance mutations not found in tissue highlights its potential value in guiding personalized treatment decisions for NSCLC patients.

## Introduction

Cancer genomics showed an unprecedented improvement during the last decade. The widespread adoption of Next-Generation Sequencing (NGS) technologies at relatively low costs, combined with the availability of an increasing number of selective drugs, has led to a paradigm shift in oncology, thus leading to significant improvements in terms of patients’ outcome.^1-6^ Given the increasing number of druggable alterations, NGS-based multi-gene testing represents the recommended approach for simultaneous detection of multiple gene alterations in a variety of solid malignancies, including non-small cell lung cancer (NSCLC), colorectal, prostatic and biliary cancer.^7,8^

Liquid biopsy (LB) offers a non-invasive method for identifying tumor genomic aberrations. This approach addresses several limitations associated with traditional tumor tissue biopsies, such as feasibility, patient discomfort and morbidity.^5,9-11^

In this context, NSCLC represents a model for LB application. In 2013, it was first demonstrated that LB could detect resistance mutations to first- and second-generation EGFR inhibitors (EGFR T790M),^12^ showing the clinical utility of LB for patients.^13^ On June 2016, the US Food and Drug Administration (FDA) approved the first plasma-based molecular assay, the Cobas *EGFR* Mutation Test 2 (Roche Molecular Systems, Inc.), a real-time polymerase chain reaction (PCR) test for the detection of *EGFR* exon 19 indels and the L858R mutations to identify NSCLC eligible for erlotinib.^14^ Recently, LB tests have consistently been proven to represent a valuable tool in guiding treatment options for metastatic patients when obtaining diagnostic tissue material is challenging.^15^ In particular, ESMO Clinical Practice Guidelines recommend the use of ctDNA testing in treatment-naive patients in which tissue biopsy is risky or contraindicated, and in pre-treated oncogene addicted patients for the detection of resistance mutations.^16-18^

In recent years, several comprehensive NGS panels have been developed and commercialized, allowing an exhaustive characterization of tumor mutational landscape, including the detection gene mutations, fusions, copy number variation (CNV), tumor mutational burden (TMB), microsatellite instability (MSI), loss of heterozygosity (LOH) and homologous recombination.^19^

These data provide a strong rationale to extend comprehensive genomic profiling (CGP) to LB in clinical practice. To this aim, we designed a head-to-head comparison of two LB CGP assays, i.e. Guardant360 CDx and TruSight Oncology 500 ctDNA, in a consecutive case cohort of NSCLS patients with known actionable targets. G360 is a FDA-approved, in service, DNA-based NGS panel, investigating single nucleotide variants (SNV), indels, gene fusions and CNV in up to 74 cancer-related genes, which has been extensively validated through numerous studies and clinical trials, showing its effectiveness in identifying actionable mutations and guiding targeted therapy in NSCLC and other cancer types.^20^

## Material and methods

### Patients' cohort

This is a prospective observational study conducted between May 2022 and March 2024, enrolling 36 consecutive male and female patients over 18 years of age with locally advanced/metastatic NSCLC who provided informed consent, which were molecularly characterized on bioptic samples prior to target therapy initiation. At disease progression, plasma samples were collected from all patients included in the study for ctDNA analysis with both TruSight Oncology 500 ctDNA version 1 (TSO500, Illumina Inc, San Diego, CA, USA) and Guardant360 CDx (G360, Guardant Health, Palo Alto, CA, USA). Whenever feasible, patients also underwent a tissue core biopsy of one of the metastatic deposits. Therapeutic decisions were undertaken based on the results obtained with the G360 LB results. The study was approved by the Internal Audit Committee and Ethics Committee (INT 283-21).

### cfDNA extraction, next-generation sequencing and data analysis

Plasma was obtained from whole blood collections (2 × 10 mL Streck tubes per assay, total volume = 20 mL). G360 is a comprehensive LB test detecting SNVs, indels, CNAs, and fusions across 74 genes in ctDNA, serving as a companion diagnostic (CDx) for multiple targeted therapies (EGFR, ERBB2, KRAS mutations) in NSCLC.^14^ Blood samples allocated for G360 were promptly shipped to Guardant Health facilities, and molecular reports delivered within 7-10 business days. The TSO500 is a LB NGS test for in-house use, designed to detect point mutations (SNV), indels and CNVs across 523 cancer-related genes, as well as fusions and splicing variants in 55 genes, and to characterize key immuno-oncology gene signatures, including TMB and MSI. After double centrifugation, cell-free DNA (cfDNA) was isolated using the QIAamp Circulating Nucleic Acid Kit (cat. 55114; Qiagen, Hilden, Germany). cfDNA was quantified using the Qubit Fluorometer 3.0 (Thermofisher, Waltham, MA, USA) and the dsDNA HS Assay (quantification range: 10 pg/µL–100 ng/µL; Thermofisher). The cfDNA fragment size was determined with the Cell-free DNA ScreenTape Analysis on TapeStation (Agilent Technologies, Inc., Santa Clara, CA). Briefly, at least 30 ng of cfDNA samples were used to generate libraries with TSO500 ctDNA KIT (Illumina). Libraries were quality-controlled with Qubit and TapeStation. Sequencing was carried out on the Illumina NovaSeq 6000, (800 million reads per sample). Raw data were analyzed using the Dragen TruSight Oncology 500 ctDNA v2.1.1 on an Illumina DRAGEN server v4. All variants that passed manufacturer’s quality check (“PASS” tag in VCF files) were used for further analyses. Polymorphisms were filtered by using publicly available databases (e.g. dbSNP, GnomAD, 1000Genomes). Variants were examined using OpenCravat^21^ and clinically annotated using ClinVar^22^ and cBio portal database^23^; variants classified as benign or likely benign were excluded from further analyses. The Integrative Genomic Viewer IGV tool^24^ was used for final check. Clinical actionability of pathogenic and likely pathogenic (P/LP) variants was defined according to the ESMO Scale for Clinical Actionability of molecular Targets (ESCAT).^25^ The final reported data were discussed by the institutional Molecular Tumor Board.^19^ The FDA-approved G360 assay was used as a reference to evaluate the analytical performance of TSO500. For comparative analysis we considered the 74 genes shared between TSO500 and G360 tests. For variants detected by G360 but not confirmed by TSO500, the relevant genomic regions were verified to be included in the TSO500 target design and to be adequately covered in terms of sequencing depth, and Binary Alignment Map (BAM) files were manually reviewed using the Integrative Genomics Viewer (IGV) tool. Standard packages for R software v 4.1.2 was used to explore the data and for descriptive statistics. *Complexheatmap* package in R was used to draw oncoplots of gene variants.

## Results

From May 2022 to March 2024, we enrolled 36 NSCLC patients progressing on tyrosine kinase inhibitors, previously characterized with molecular tests performed in our institution. Clinico-pathological and molecular characteristics of the study population are summarized in Table 1. In eight patients, a second blood sample was taken at further disease progression, leading to an overall number of 44 collected samples. G360 provided valid reports in 42 out of 44 cases (95.45%). In the remaining two cases, G360 results did not meet the internal quality criteria. Regarding TSO500, we obtained two 10 ml tubes for each patient, and both were used for plasma extraction (mean plasma quantity per patient: 8,73 ml, range: 6-12 ml). In 12 out of 44 samples (27.2%), the ctDNA content was below 30 ng (median ctDNA content of unprocessed samples: 17.09 ng; range: 7-26.3 ng), and the test was therefore not performed. Notably, neither among evaluable nor non-evaluable samples was there any correlation observed between the volume of plasma used for extraction (mean: 8.73 mL; range: 6.00–12.00 mL) and the total amount of cfDNA obtained (Pearson’s r = 0.18), suggesting that input volume alone did not account for differences in ctDNA yield.

**Table 1.** Patient characteristics.

	Patients enrolled (n=36)	Comparative analysis patients (n=27)
**Gender**		
Male	7 (19.4%)	6 (22.2%)
Female	29 (80.5%)	21 (77.8%)
**Age (years)**		
Mean ± SD	64.6 ± 10.7	63.1 ± 10.02
Median (range)	65.7 (37.8-83.3)	60.7 (44.2-83.3)
**Smoking history**		
Yes	18 (50%)	14 (51.8%)
No	18 (50%)	12 (44.4%)
**Previous oncological surgery**		
Yes	11 (30.5%)	7 (25.9%)
No	25 (69.4%)	20 (74.07%)
**Histology**		
Adenocarcinoma	33 (91.6%)	25 (92.5%)
Large Cell Neuroendocrine Carcinoma	2 (5.5%)	2 (7.4%)
Adenosquamous lung carcinoma	1 (2.7%)	-
**Prior molecular characterization**		
External	9 (22.2%)	5 (18.5%)
Ion AmpliSeq Cancer Hotspot Panel (CHP)	6 (16.6%)	4 (14.8%)
DNA Oncomine Research Assay Plus	10 (27.7%)	8 (29.6%)
Targeted panel - LKB1	7 (19.4%)	7 (25.9%)
Real Time PCR	5 (13.8%)	3 (11.1%)
RNA Oncomine Research Assay Plus	16 (44.4%)	12 (44.4%)
Archer FusionPlex Lung Panel	2 (5.5%)	2 (7.4%)
Fluorescent In Situ Hybridization (FISH)	18 (50%)	13 (48.1%)
**Driver molecular alteration**		
***EGFR***	20 (55.5%)	15 (55.5%)
***ALK***	9 (25%)	5 (18.5%)
***ROS1***	3 (8.33%)	3 (11.1%)
***RET***	3 (8.33%)	3 (11.1%)
***BRAF***	2 (5.55%)	2 (7.4%)
**Line of therapy**		
I	26 (72.2%)	20 (74.04%)
II	6 (16.6%)	3 (11.1%)
>II	4 (11.1%)	4 (14.8%)
**Latest therapy**		
TKI inhibitors	33 (91.6%)	24 (88.8%)
Immunotherapy	1 (2.7%)	1 (3.7%)
Chemotherapy	2 (5.5%)	2 (7.4%)
**Previous Radiotherapy**		
Yes	25 (69.4%)	18 (66.6%)
No	11 (30.5%)	9 (33.3%)

Therefore, the comparative analysis was performed on 31 samples from 27 patients for which paired G360 and TSO500 data were available (Table 1). Study design is summarized in Figure 1.

**Figure 1.**
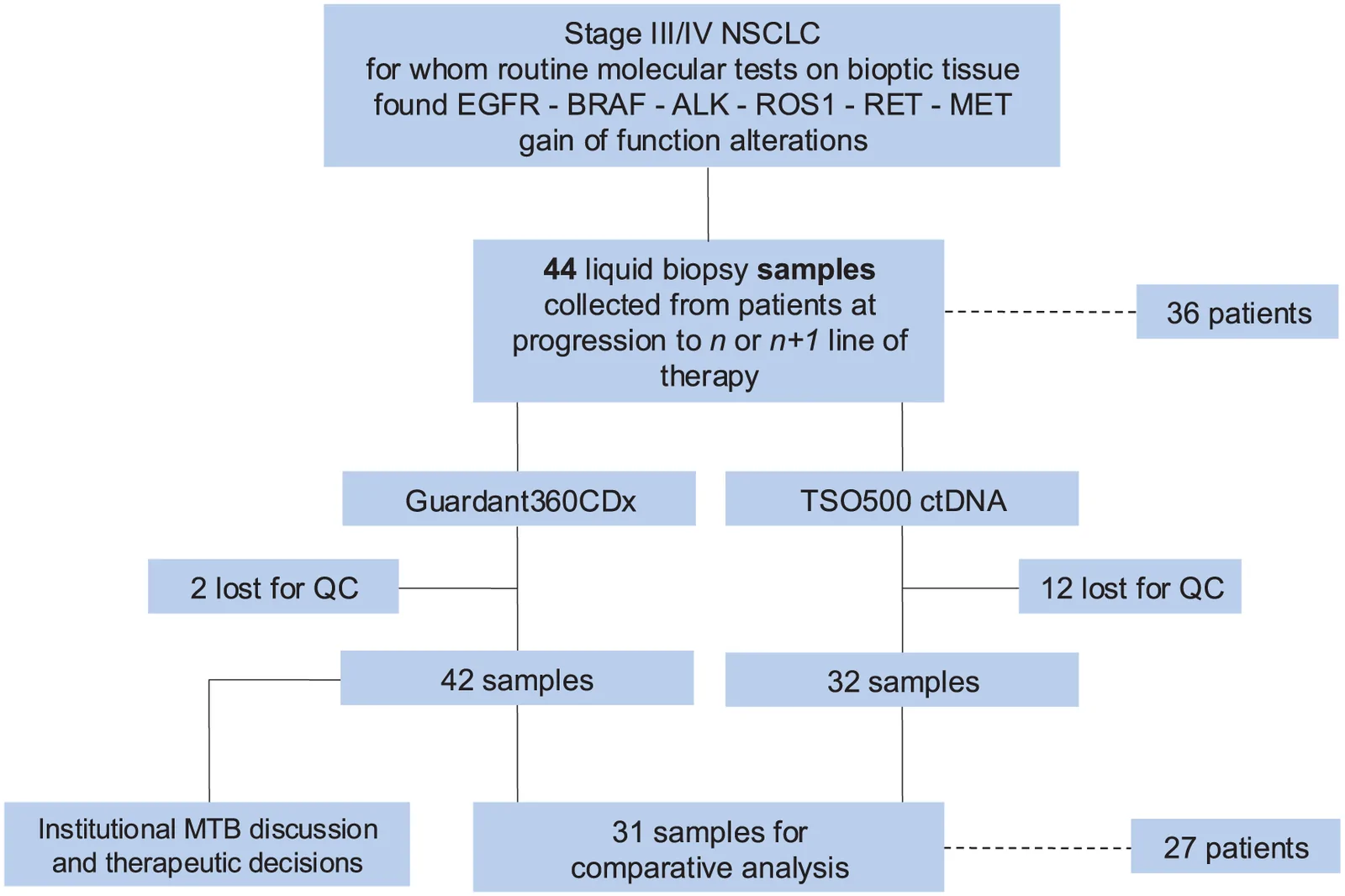
Study design.

### G360 and TSO molecular results

Among the 31 samples included in the comparative analysis, G360 identified 137 variants (mean per patient: 4.42, range 0-15). Ninety-five variants were annotated as P/LP (mean per patient: 3.06, range: 0-9), and 42 as variants of unknown significance (VUS; mean per patient: 1.35, range: 0-9). In particular, G360 identified 117 SNV/indels, five gene fusions and 15 CNVs. TSO500 ctDNA assay identified 723 variants (mean per patient: 23.32, range: 7-123). Two hundred and seventeen variants were P/LP (mean per patient: 7, range: 0-27), and 489 VUS (mean per patient: 15.77, range: 5-96), including 598 SNV/indels, five fusions and 103 CNV. No significant correlation was found between the DNA input and the number of variants obtained (Pearson’s r = -0.017, p = 0.92) (Online Supplementary Figure S1A). The mean value of TMB obtained by TSO 500 ctDNA assay was 8.9 mut/Mb (range: 0–73.4). No significant correlation was found between the DNA input and TMB value (Pearson’s r = 0.132, p = 0.47) (Online Supplementary Figure S1B). No significant association was found between ctDNA content and number of metastatic sites (Wilcoxon rank-sum test, p = 0.43) (Online Supplementary Figure S2).

### G360 and TSO500 comparative analysis

Overall, TSO500 correctly identified 111 out of the 137 variants (81.02%) detected by G360. TSO500 found 102 variants (range per sample: 1-19, median: 3) which were not identified by G360, leading to an overall specificity of 0.97 (range: 0.74-1) (Figure 2A and 2B). TSO500 correctly identified 81 out of the 95 P/LP variants (85.26%) found by G360 (median sensitivity 0.89, range 0.33-1). Additionally, TSO500 detected 138 P/LP variants (range: 1-22, median: 3) not identified by G360 (specificity 0.99, range 0.88-1) (Figure 2C and 2D).

**Figure 2.**
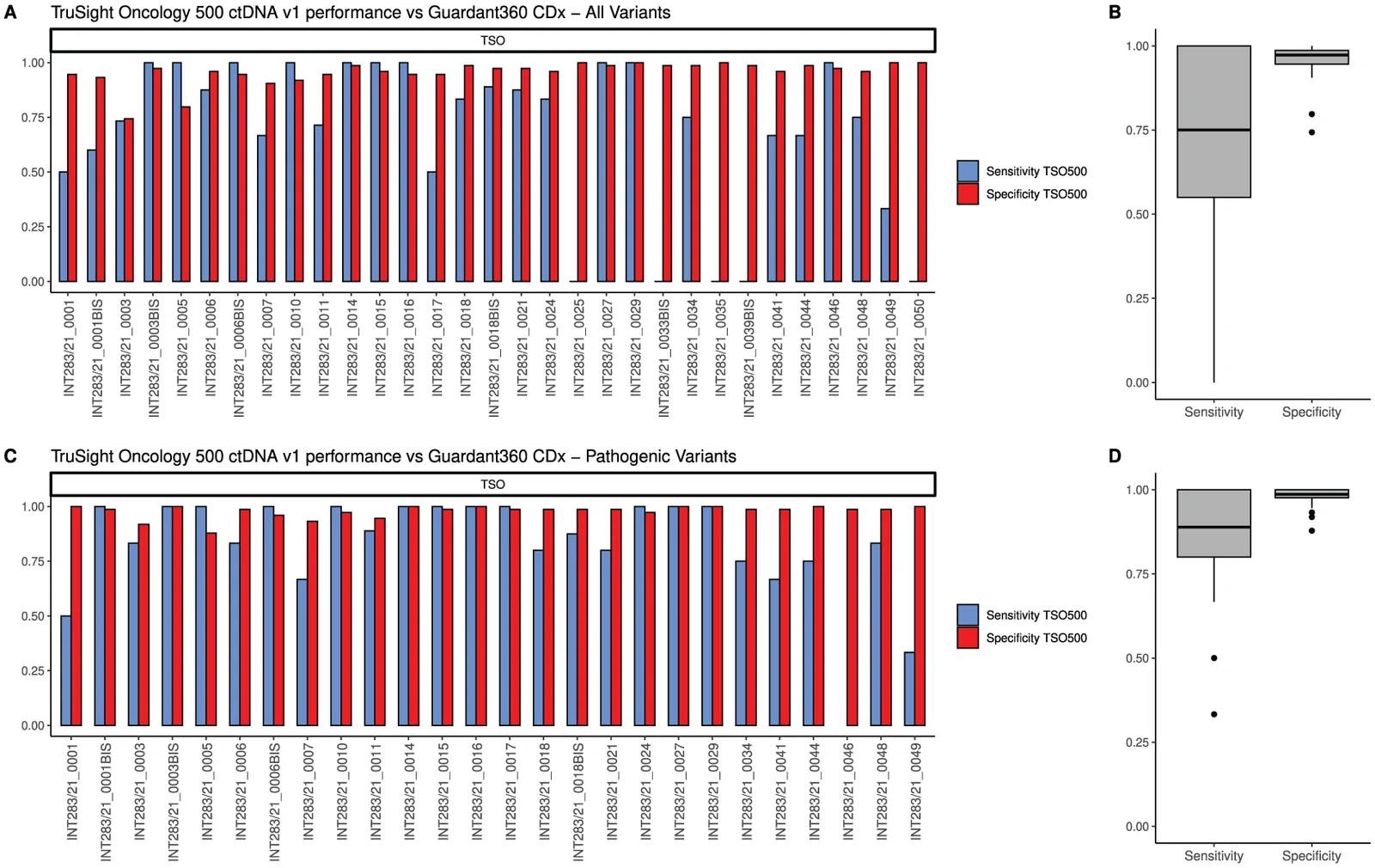
Analytical performance of the TruSight Oncology 500 ctDNA v1 (TSO500) platform compared to the G360360 CDx test, in evaluating all variants (A,B) and pathogenic/likely pathogenic variants (C,D). The bar charts (A,C) display the proportion of correctly identified variants (sensitivity, blue) and correctly not identified (specificity, red) of TSO500 for each sample. Samples INT283/21_0025 and INT283/21_0035 resulted wild type by G360 testing, and therefore sensitivity calculation was not applicable. As well, sensitivity cannot be extracted in sample INT283/21_0046 where G360 did not identify pathogenic variants. Boxplots (B,D) show the sensitivity and specificity distributions across the whole dataset.

### Comparative analysis through ESCAT tier classification

For the evaluation of the potential clinical impact, we compared alterations identified by TSO500 and G360 across ESCAT classes. TSO500 and G360 were consistent in detecting ESCAT I (20 and 21 variants by TSO500 and G360, respectively) and ESCAT II variants (three and one variants, respectively), while TSO500 identified a higher number of ESCAT III (17 and 12 variants) and ESCAT IV (30 and nine variants) variants (Figure 3). Compared with G360, TSO500 showed high sensitivity across ESCAT classes (95.2%, 100%, 58.3%, 88.8% for ESCAT I, II, III, IV class mutations, respectively).

**Figure 3.**
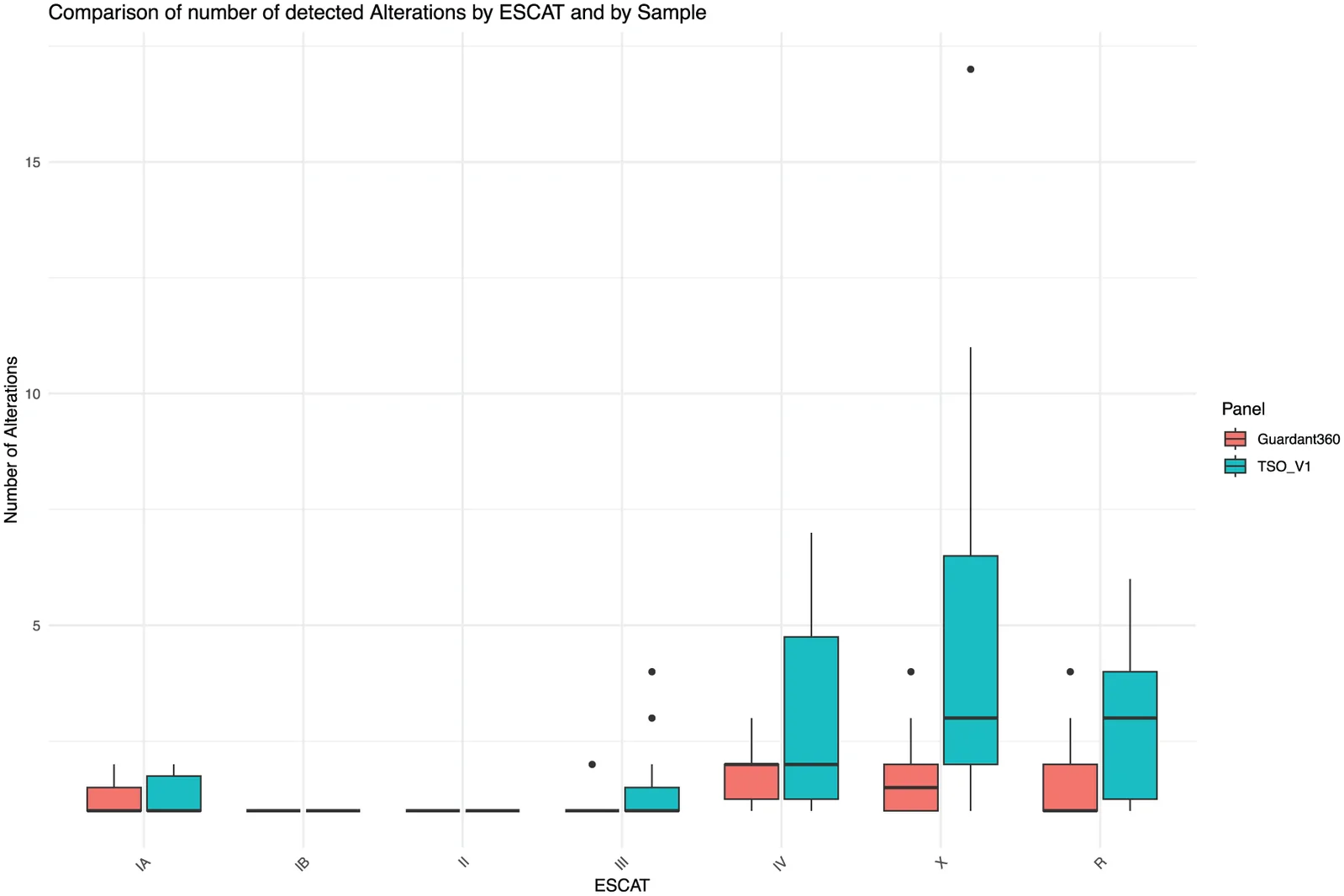
Boxplots showing the distribution of the absolute number of variants detected in each sample across ESCAT classes.

In particular, among standard of care actionable targets (ESCAT I), TSO500 did not detect an *EGFR* mutation (p.Leu858Arg) that was identified by G360; in this case, TSO500 run showed a low median coverage (927) (Figure 4).

**Figure 4.**
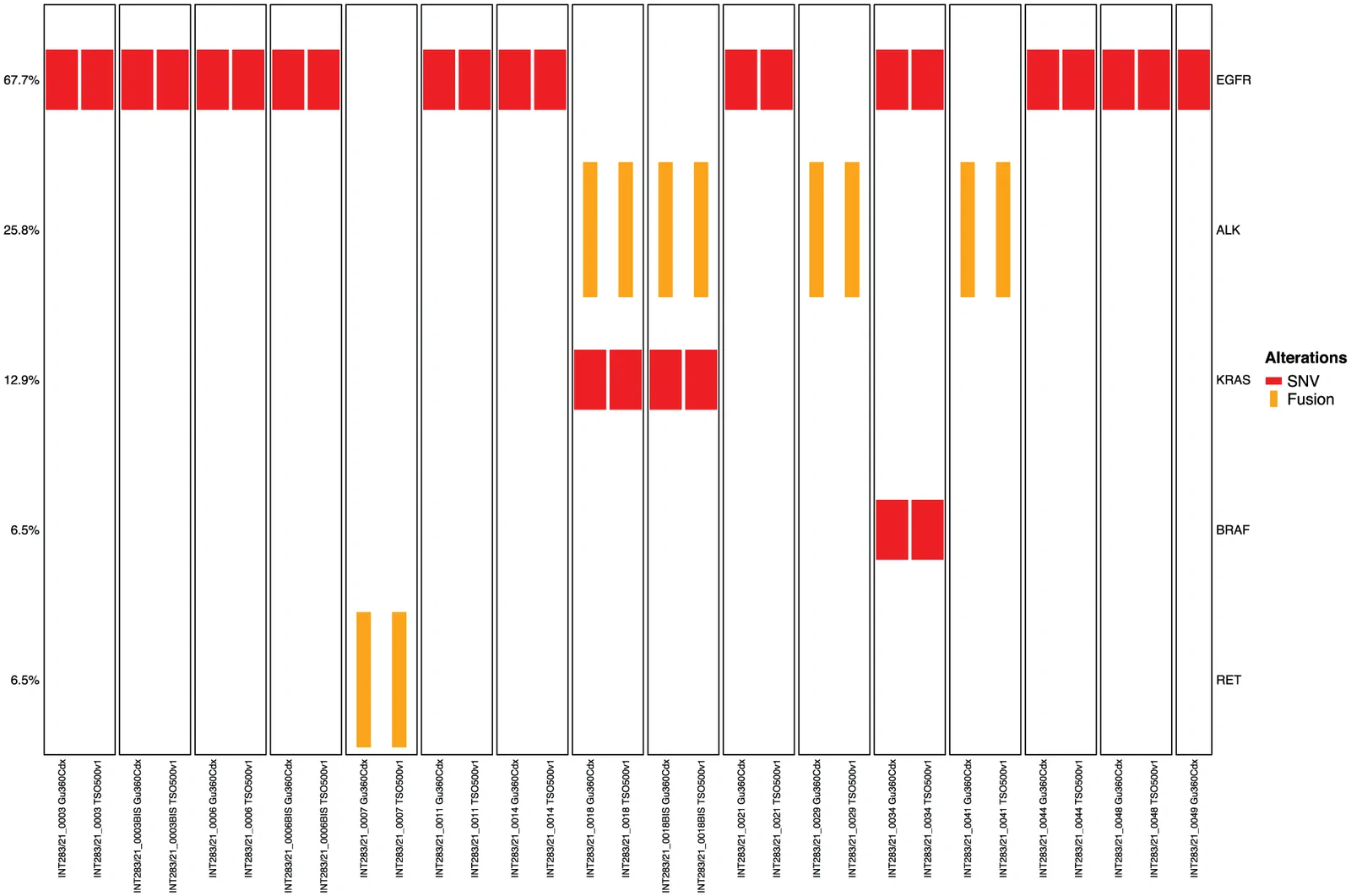
Oncoplot showing therapeutically actionable variants (ESCAT I) in EGFR, ALK, KRAS (p.G12C) and RET genes, detected by G360 (left side of each column) and TSO500 (right side of each column). Single nucleotide and indels variants are represented by short red rectangles, while gene fusions by long yellow rectangles.

Notably, all five gene fusions identified by G360 (four ALK and one RET fusions) were confirmed by TSO. TSO500 did not detect five ESCAT IIIA variants, including two *PIK3CA*, one *BRCA1*, one *PTEN* and one *ATM* variants. Except for the *BRCA1* mutation (VAF: 8%), all these variants were characterized by a low variant allele frequency in G360 (range: 0.05%-0,38%). Of note, after a subsequent review of the BAM files using the IGV software, one of the *PIK3CA* variants and the *PTEN* variant were detected by TSO500, albeit at low frequency (*PIK3CA*, VAF: 0.07%) or affected by systematic noise (*PTEN*, VAF: 0.24). These variants had been excluded from the VCF files due to pre-specified quality filtering. On the other hand, no additional ESCAT I mutations missed by G360 were identified by TSO500 (Figure 4). A high sensitivity (14/15, 93,3%) was also found with regard to CNV for *EGFR* (six cases), *BRAF* (two), *CCNE1* (two), *MET* (one), *KRAS* (one) and *CCND1* (one). TSO500 identified 44 potential intrinsic or acquired resistance mutations in 12 out of 26 patients (46.2%), compared to 25 resistance mutations detected by G360 in 12 patients, including *EGFR* p.L718G, p.C797S and amplification, *PIK3CA* mutations, *MET* amplification, cell cycle genes alterations, *FGF* genes alterations and *RAS* genes alterations.^20,26-32^ In particular, TSO500 detected all but three resistance mutations identified by G360, including the two aforementioned *PIK3CA* mutations in two patients treated with anti-EGFR therapy, and one *ALK* mutation (p.Gly1202Arg) in a patient with *ALK* fusion treated with lorlatinib.

Finally, we observed a high correlation in variant allele frequencies among gene alterations detected by both TSO and G360 (Sperman ρ = 0.935, CCC=0.993) (Online Supplementary Figure S3).

### Comparison between LB and tissue NGS results at relapse

In nine out of 27 patients (33.3%), concurrent tissue-based CGP was performed at the same disease progression timepoint, allowing a direct comparison between tissue and LB NGS results.

Overall, in 7/9 cases TSO500 identified 16 potentially actionable (ESCAT I-IV) and resistance mutations which were not identified by concurrent tissue NGS, including two ESCAT IA variants (two *KRAS* G12C), five ESCAT IIIA variants (two *BRCA1* deletions, one *PIK3CA* mutation, one *KRAS* mutation and one *KMT2A* mutation), one ESCAT IIIB (MET mutation), five ESCAT IV variants (one *CHEK2* mutation, and *PIK3CA*, *FGFR3*, *BRAF*, and CDK6 amplifications), and three EGFR resistance alterations (one amplification and two mutations). Of note, in a patient with an *ALK*-*EML4* positive NSCLC progressing after two lines of ALK inhibitors (Alectinib and Lorlatinib), both G360 and TSO500 detected a *KRAS* p.G12C and a *PIK3CA* p.E542K mutation, which were not identified by concurrent tissue NGS. Upon further progression to a third line of treatment with carboplatin-pemetrexed, this patient was subjected to additional molecular profiling in both tissue and LB samples. The *KRAS* variant was detected in subsequent analysis both in tissue and LB samples, while the *PIK3CA* variant was identified in the plasma sample but not in tissue. For the complete list of pathogenic mutations detected by TSO500, G360 and tissue NGS see Online Supplementary Table S1.

### Comparison between LB at relapse and tissue NGS at diagnosis

Based on the availability of tissue NGS at diagnosis, we evaluated whether the resistance mechanisms identified by TSO500 LB were intrinsic or acquired. Comparable data for pre-treatment tumor tissue and LB at relapse were available for 5/12 patients (the remaining seven patients were characterized in tissue at diagnosis by targeted small NGS panels). In four of these cases, TSO500 LB identified mutations which were not detected in tissue at diagnosis, thus representing putative acquired mutations. These included acquired *EGFR* p.L718Q and *BRAF* p.V600E mutations in two patients with *EGFR* mutations treated with osimertinib, receiving platinum-based chemotherapy and savolitinib plus osimertinib/placebo within a clinical trial (NCT03778229), respectively; one *MET* amplification plus *KRAS* p.G12C mutation in an *ALK* positive patients previously treated with alectinib, receiving platinum based chemotherapy; and one *BRAF* p.V600E plus *ALK* missense mutation in two *ALK*-rearranged patients treated with lorlatinib. However, in patients harboring gene alterations with strong evidence of actionability (*BRAF* p.V600E and *KRAS* p.G12C) detected by LB, targeted therapy remained inaccessible due to the unavailability of tissue-based NGS confirmation for *BRAF* and limited off-label access or clinical trial availability for *KRAS* p.G12C. This highlights the critical need for broader drug access for alterations identified through LB.

In the remaining patient, TSO500 LB did not detect any acquired variant, confirming the *ERBB2*, *CCNE1*, and *EGFR* amplifications previously identified in the pre-treatment tissue biopsy by CGP.

At baseline, 11 patients had actionable gene fusions. At disease progression, LB detected gene fusions in four of these patients (five out of 12 plasma samples, 41.6%).

In the remaining seven patients, G360 testing also did not detect any gene fusion. Concurrent tissue biopsies at progression were available for three of these seven patients; in two cases, tissue analysis confirmed the persistence of the original *RET* fusion, while in the third case, both IHC and ISH confirmed the loss of the *ALK* fusion.

Among all tissue samples collected at progression, gene fusions were detected in seven cases. TSO500 assay successfully identified gene fusions in five of these cases, yielding a detection rate of 71.4%.

Comparative analysis between TMB values detected by tissue CGP (both prior target therapy and at disease progression) and LB TSO500 revealed poor correlation (Pearson’s r = 0.228, p = 0.498) (Online Supplementary Figure S4).

## Discussion

Our study provides the first real-world evaluation of the TSO500 ctDNA assay compared to the FDA-approved Guardant360 platform, demonstrating robust analytical performance and potential clinical utility in NSCLC patients progressing on targeted therapies.

We observed a high concordance between TSO500 and G360 in detecting clinically actionable alterations, particularly for ESCAT I variants (sensitivity: 95.2%), in line with data previously reported by Woodhouse et al. who demonstrated >95% concordance between FoundationOne Liquid CDx and different orthogonal methods in detecting actionable variants.^33^

Importantly, our findings suggest that TSO500's capabilities, combined with its cost-effectiveness in high-volume institutional settings, position in-house liquid biopsy CGP as a viable alternative for cancer centers with adequate infrastructure, volumes and expertise, to commercial and more expensive externalized services.

A key advantage of the comprehensive TSO500 panel lies in its ability to detect a broad spectrum of genomic alterations, including a significant number of ESCAT III and IV variants. While ESCAT I alterations guide standard-of-care therapies, lower-tier variants often hold relevance for patients who have exhausted approved treatment options. The identification of these additional alterations may facilitate patient enrollment in clinical trials or support off-label targeted therapy access, offering potential therapeutic strategies beyond conventional treatments.

The sensitivity of TSO500 in detecting gene fusions is particularly noteworthy. While RNA-based panels are traditionally preferred, especially in tissue NGS, DNA-based LB tests also show a robust analytical validity.^34^ González-Medina et al. demonstrated the effectiveness of the VHIO-iCCA custom NGS panel in monitoring *FGFR2* fusion-positive patients during therapy, showing high sensitivity by detecting 16 fusions in plasma samples from 18 cholangiocarcinoma patients (sensitivity: 88.9%) with known *FGFR* fusions.^35^ Similarly, Kasi and colleagues reported a high sensitivity of ctDNA testing for identifying actionable fusions across 53,842 patients profiled with FoundationOneLiquid CDx.^36^ Conversely, in a comprehensive analysis of matched tissue and plasma samples from NSCLC patients, Lin et al. demonstrated that a 168 genes commercial LB NGS panel detected 67% (35/52) of fusions previously identified through RNA-based tissue NGS.^37^ Although based on a small series, our study confirms these findings for the TSO500 LB assay, that was able to detect five out of seven (71.4%, including 3 *ALK*-*EML4* and 2 *KIF5B*-*RET*) gene fusions identified by concurrent tissue NGS.

TMB measured by TSO500 did not show a strong correlation with tissue TMB, a finding consistent with other studies.^38^ While TMB estimation from ctDNA is technically feasible and has shown potential correlation with immunotherapy response in certain clinical contexts, our findings underscore the significant limitations that currently limit its clinical utility. These include assay-specific variability, lack of standardization across platforms, and poor concordance with tissue-based TMB measurements, the current gold standard. Consequently, current American Society of Clinical Oncology and ESMO guidelines, recommend against using ctDNA-derived TMB as a sole biomarker for immunotherapy selection without tissue confirmation. These technical and interpretive challenges highlight the need for further analytical and clinical validation of ctDNA-based TMB assessment, which remains a priority for ongoing research in precision oncology.^39-45^

A potential flow of the TSO500 assay is represented by the relatively high output of ctDNA required for the analysis. In our study, 12 out of the 34 (27%) samples yielded less than 30 ng of DNA and were therefore not profiled in accordance with manufacturers’ guidelines. The recent release of TSO500 v2, that requires a lower (20 ng) DNA input could significantly limit the failure rate. Additionally, we found in our study that ctDNA quantity did not affect TMB values, and that SNV/InDels were detected at high sensitivity. Interestingly, using TSO500 v2 in an independent cohort of metastatic cancer patients of different histologies, allowed our group to obtain informative LB data in 89% of the cases (unpublished results).

Our study underlines the complementary value of LB in detecting therapeutic targets and resistance mutations not identified in concurrent tissue samples.^46^ In particular, TSO500 was more efficient than tissue biopsy in capturing tumor heterogeneity and emerging mutations associated with disease progression. Such findings highlight that applying CGP in LB allows capturing genomic alterations that might be missed by tissue NGS due to tumor heterogeneity in advanced disease.

Of note, in comparative NGS studies, a potential challenge is the management of discordant results, which may have implications for treatment planning. Discordant findings can be addressed through manual review of BAM files using tools such as IGV — as applied in our study, where two variants initially excluded by TSO500 quality filters were recovered upon visual inspection — and, when clinically relevant, through orthogonal validation by orthogonal methods (PCR) or discussion within a Molecular Tumor Board.

In conclusion, our findings support the analytical validity and clinical utility of TSO500 LB, demonstrating high concordance with the FDA-approved G360, high accuracy in the identification of clinically actionable variants, and robust sensitivity for gene fusions detection. These attributes position TSO500 as a robust tool in precision oncology, enabling tailored treatment decisions. Future research should focus on refining ctDNA-based TMB assessment and optimizing DNA input requirements to maximize the clinical utility of TSO500.

## Supplemental Material

sj-pdf-1-tmj-10.1177_03008916261438383 – Supplemental material for Assessing in house comprehensive genomic profiling by liquid biopsy for NSCLC patients Supplemental material, sj-pdf-1-tmj-10.1177_03008916261438383 for Assessing in house comprehensive genomic profiling by liquid biopsy for NSCLC patients by Andrea Vingiani, Alberta Piccolo, Adele Busico, Iolanda Capone, Elena Tamborini, Federica Perrone, Cinzia De Marco, Paolo Verderio, Chiara M. Ciniselli, Claudia Proto, Marta Brambilla, Giuseppe Lo Russo, Elena Conca, Andrea Devecchi, Daniele Lorenzini, Luca Agnelli and Giancarlo Pruneri in Tumori Journal

sj-pdf-2-tmj-10.1177_03008916261438383 – Supplemental material for Assessing in house comprehensive genomic profiling by liquid biopsy for NSCLC patients Supplemental material, sj-pdf-2-tmj-10.1177_03008916261438383 for Assessing in house comprehensive genomic profiling by liquid biopsy for NSCLC patients by Andrea Vingiani, Alberta Piccolo, Adele Busico, Iolanda Capone, Elena Tamborini, Federica Perrone, Cinzia De Marco, Paolo Verderio, Chiara M. Ciniselli, Claudia Proto, Marta Brambilla, Giuseppe Lo Russo, Elena Conca, Andrea Devecchi, Daniele Lorenzini, Luca Agnelli and Giancarlo Pruneri in Tumori Journal

sj-pdf-3-tmj-10.1177_03008916261438383 – Supplemental material for Assessing in house comprehensive genomic profiling by liquid biopsy for NSCLC patients Supplemental material, sj-pdf-3-tmj-10.1177_03008916261438383 for Assessing in house comprehensive genomic profiling by liquid biopsy for NSCLC patients by Andrea Vingiani, Alberta Piccolo, Adele Busico, Iolanda Capone, Elena Tamborini, Federica Perrone, Cinzia De Marco, Paolo Verderio, Chiara M. Ciniselli, Claudia Proto, Marta Brambilla, Giuseppe Lo Russo, Elena Conca, Andrea Devecchi, Daniele Lorenzini, Luca Agnelli and Giancarlo Pruneri in Tumori Journal

sj-pdf-4-tmj-10.1177_03008916261438383 – Supplemental material for Assessing in house comprehensive genomic profiling by liquid biopsy for NSCLC patients Supplemental material, sj-pdf-4-tmj-10.1177_03008916261438383 for Assessing in house comprehensive genomic profiling by liquid biopsy for NSCLC patients by Andrea Vingiani, Alberta Piccolo, Adele Busico, Iolanda Capone, Elena Tamborini, Federica Perrone, Cinzia De Marco, Paolo Verderio, Chiara M. Ciniselli, Claudia Proto, Marta Brambilla, Giuseppe Lo Russo, Elena Conca, Andrea Devecchi, Daniele Lorenzini, Luca Agnelli and Giancarlo Pruneri in Tumori Journal

sj-xlsx-5-tmj-10.1177_03008916261438383 – Supplemental material for Assessing in house comprehensive genomic profiling by liquid biopsy for NSCLC patients Supplemental material, sj-xlsx-5-tmj-10.1177_03008916261438383 for Assessing in house comprehensive genomic profiling by liquid biopsy for NSCLC patients by Andrea Vingiani, Alberta Piccolo, Adele Busico, Iolanda Capone, Elena Tamborini, Federica Perrone, Cinzia De Marco, Paolo Verderio, Chiara M. Ciniselli, Claudia Proto, Marta Brambilla, Giuseppe Lo Russo, Elena Conca, Andrea Devecchi, Daniele Lorenzini, Luca Agnelli and Giancarlo Pruneri in Tumori Journal

## Data Availability

The sequencing datasets used and analyzed during the current study are not publicly available due to privacy/ethical reasons. Datasets are available from the corresponding author (CC) on reasonable request and creation of a data usage agreement with our institution.
