# Supplementary material for: Assessing in house comprehensive genomic profiling by liquid biopsy for NSCLC patients
Source: Tumori. 2026 Jun 24;112(5):413–22. doi: 10.1177/03008916261438383 (PMC13687596; doi:10.1177/03008916261438383)
Supplement: Supplementary file 1 [file sj-pdf-1-tmj-10.1177_03008916261438383.pdf]

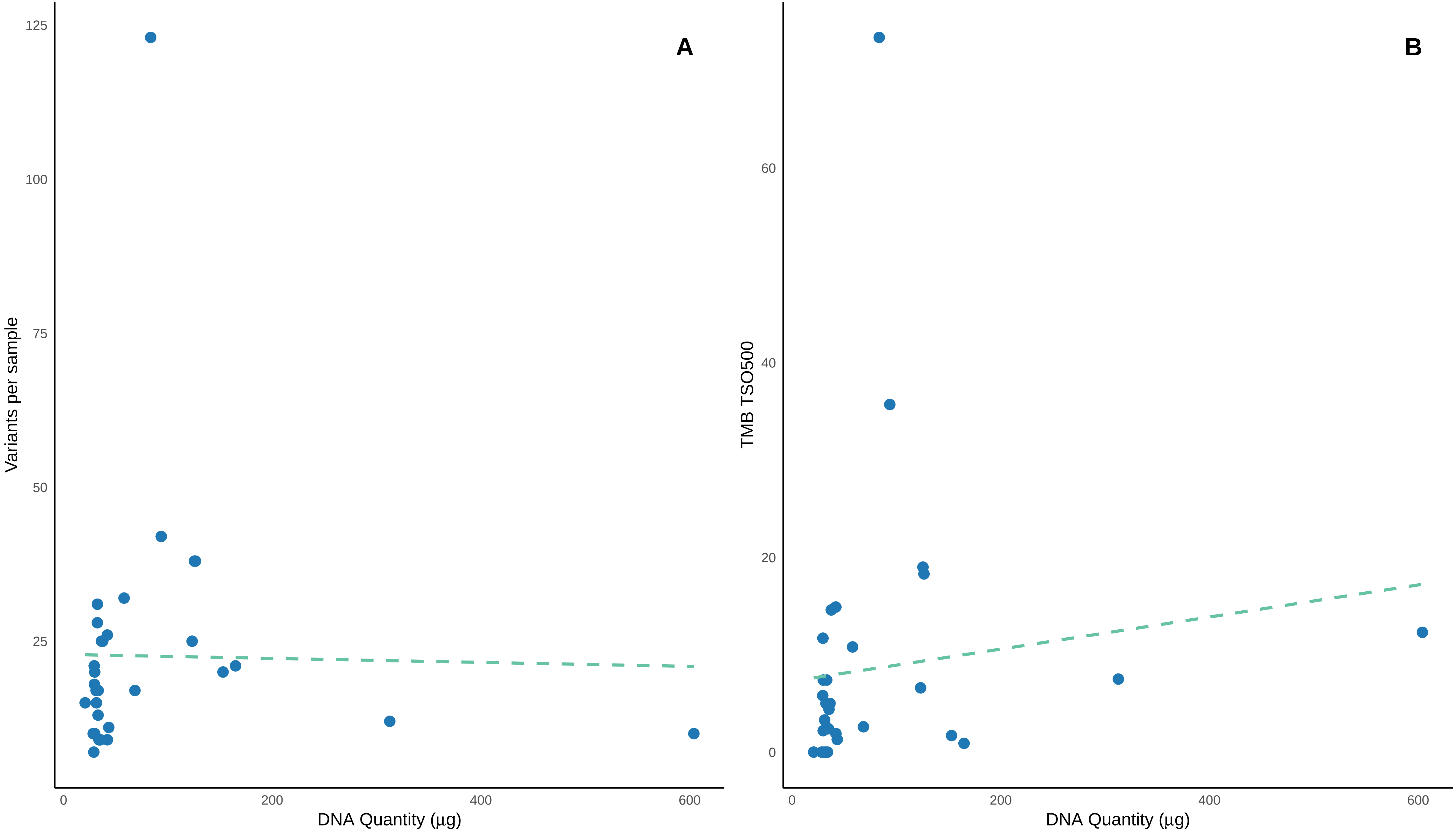

A
B
Variants per sample
TMB TSO500
125
100
75
50
25
60
40
20
0
0
200
400
600
DNA Quantity (µg)
DNA Quantity (µg)
