# Supplementary material for: Assessing in house comprehensive genomic profiling by liquid biopsy for NSCLC patients
Source: Tumori. 2026 Jun 24;112(5):413–22. doi: 10.1177/03008916261438383 (PMC13687596; doi:10.1177/03008916261438383)
Supplement: Supplementary file 2 [file sj-pdf-2-tmj-10.1177_03008916261438383.pdf]

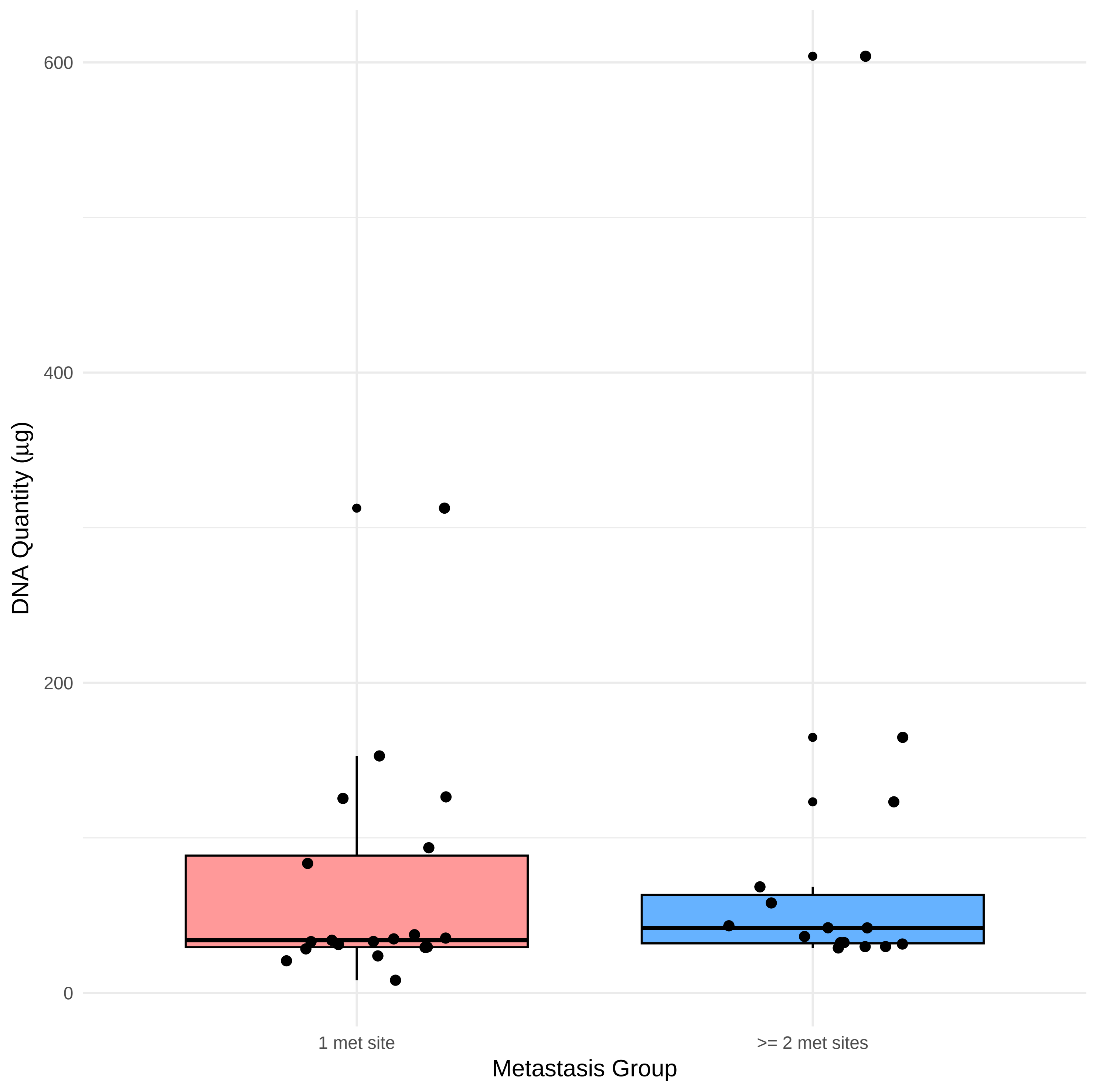

600
400
200
0
DNA Quantity (µg)
1 met site
>= 2 met sites
Metastasis Group
