# Supplementary material for: Assessing in house comprehensive genomic profiling by liquid biopsy for NSCLC patients
Source: Tumori. 2026 Jun 24;112(5):413–22. doi: 10.1177/03008916261438383 (PMC13687596; doi:10.1177/03008916261438383)
Supplement: Supplementary file 3 [file sj-pdf-3-tmj-10.1177_03008916261438383.pdf]

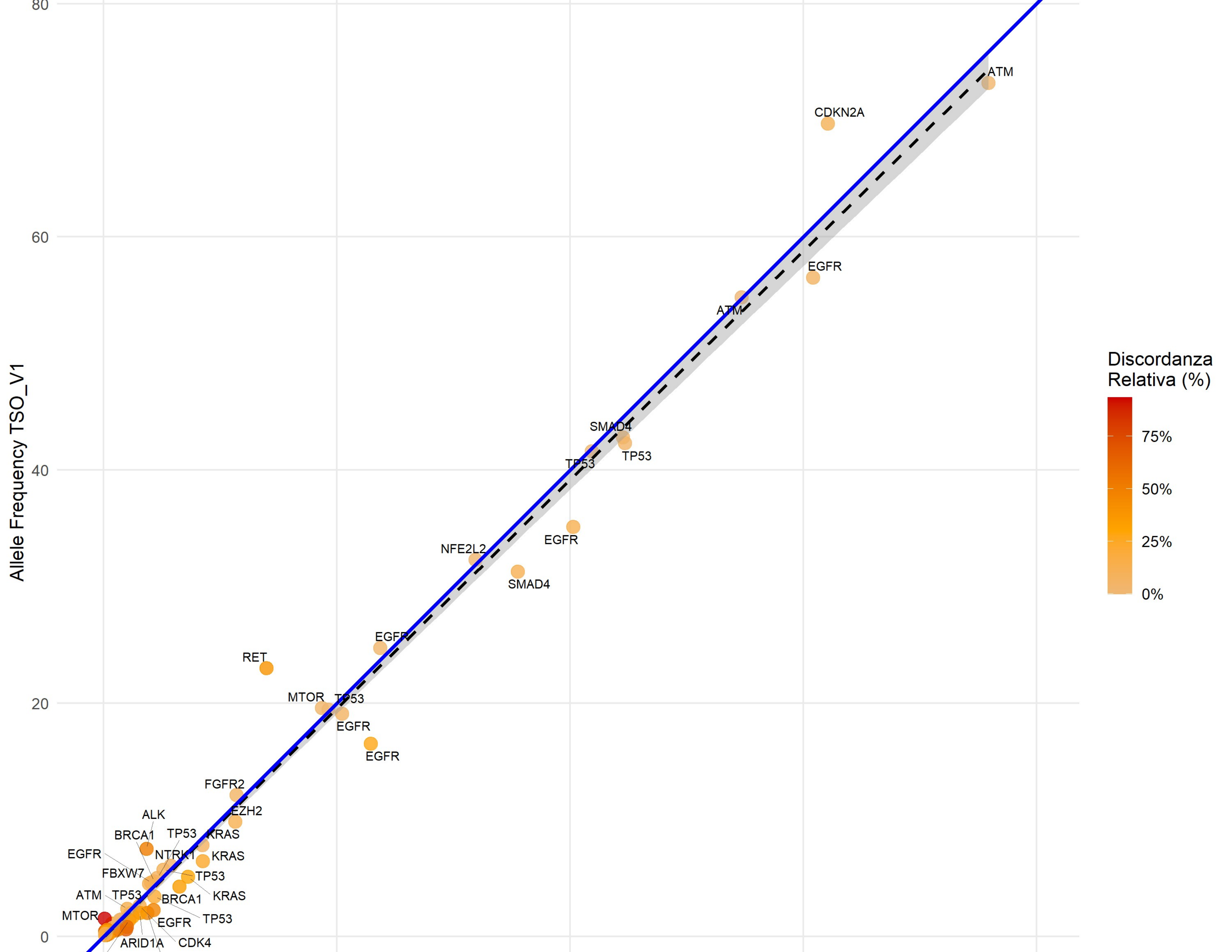

80
60
40
20
0
Allele Frequency TSO_V1
Discordanza
Relativa (%)
75%
50%
25%
0%
ATM
CDKN2A
EGFR
ATM
SMAD4
TP53
TP53
EGFR
NFE2L2
SMAD4
EGFR
RET
MTOR
TP53
EGFR
EGFR
FGFR2
EZH2
ALK
BRCA1
TP53
KRAS
EGFR
NTRK1
KRAS
FBXW7
TP53
ATM
TP53
BRCA1
KRAS
MTOR
TP53
EGFR
ARID1A
CDK4
