# Supplementary material for: Assessing in house comprehensive genomic profiling by liquid biopsy for NSCLC patients
Source: Tumori. 2026 Jun 24;112(5):413–22. doi: 10.1177/03008916261438383 (PMC13687596; doi:10.1177/03008916261438383)
Supplement: Supplementary file 4 [file sj-pdf-4-tmj-10.1177_03008916261438383.pdf]

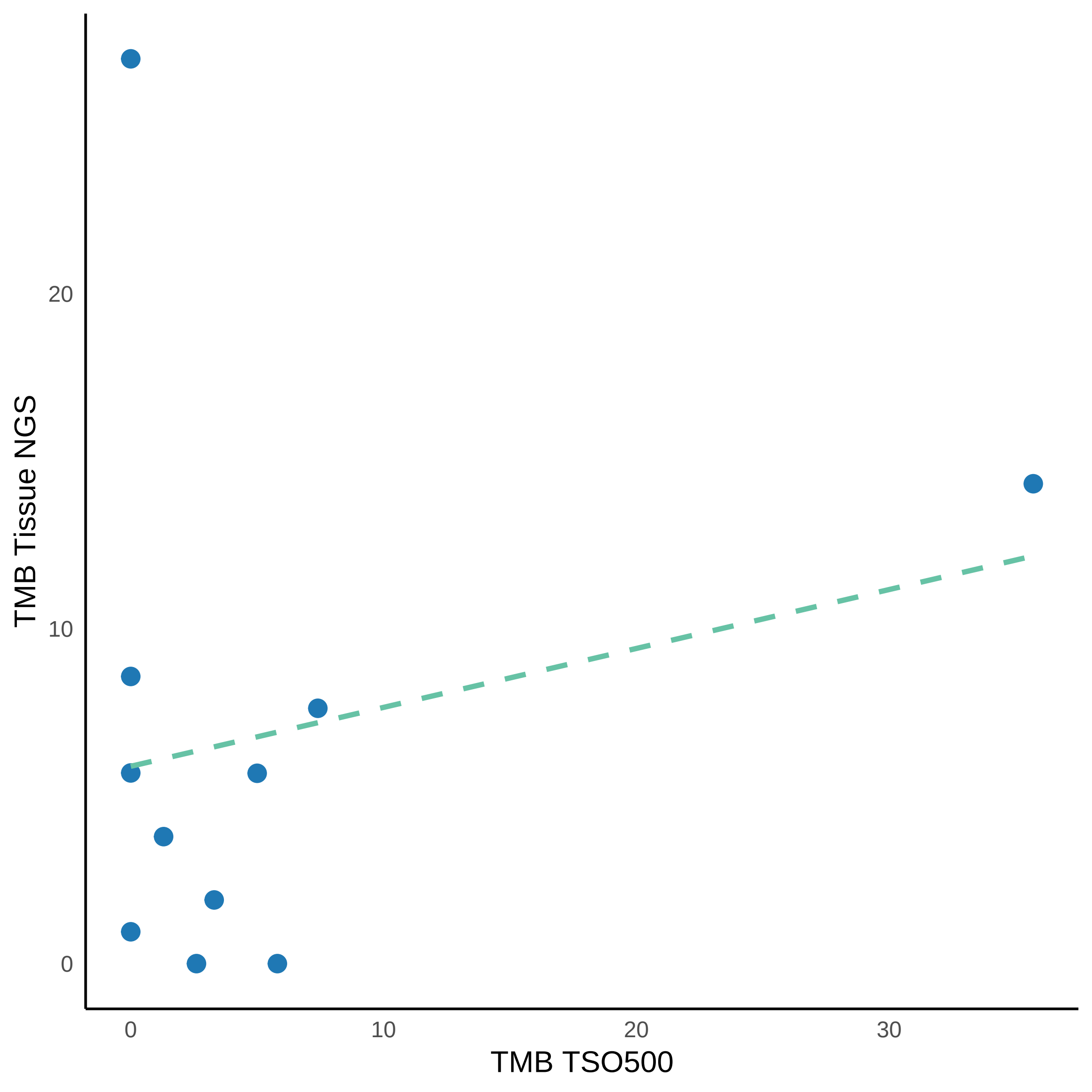
TMB Tissue NGS
20
10
0
0
10
20
30
TMB TSO500
